# Religious meaning system and life satisfaction: the mediating role of meaning in life among Polish people with multiple sclerosis

**DOI:** 10.3389/fpsyt.2023.1352021

**Published:** 2024-01-11

**Authors:** Maciej Wilski, Marcin Wnuk, Waldemar Brola, Małgorzata Szcześniak, Marek Żak, Piotr Sobolewski, Katarzyna Kapica-Topczewska, Joanna Tarasiuk, Agata Czarnowska, Alina Kułakowska, Beata Zakrzewska-Pniewska, Halina Bartosik-Psujek, Katarzyna Kubicka-Bączyk, Natalia Morawiec, Monika Adamczyk-Sowa, Adam Stepien, Zaborski Jacek, Anna Ratajczak, Marcin Ratajczak, Roman Szałachowski, Zdzisław Kroplewski, Beata Lech, Adam Perenc, Małgorzata Popiel, Andrzej Potemkowski

**Affiliations:** ^1^Department of Adapted Physical Activity, Poznan University of Physical Education, Poznan, Poland; ^2^Department of Psychology, Adam Mickiewicz University, Poznań, Poland; ^3^Collegium Medicum, Jan Kochanowski University, Kielce, Poland; ^4^University of Szczecin, Szczecin, Poland; ^5^Department of Neurology, Medical University of Bialystok, Bialystok, Poland; ^6^Department of Neurology, Medical University of Warsaw, Warsaw, Poland; ^7^College of Medical Sciences, University of Rzeszow, Rzeszow, Poland; ^8^Department of Neurology, Medical University of Silesia, Katowice, Poland; ^9^Military Institute of Medicine (Poland), Warsaw, Poland; ^10^Department of Neurology and Neurorehabilitation, Międzyleski Specialist Hospital, Warsaw, Poland; ^11^Pomeranian Medical University, Szczecin, Poland; ^12^Clinical Trial Center for MS-Patients, Szczecin, Poland; ^13^Clinical Provincial Hospital No. 2 im. St. Jadwiga Królowej in Rzeszów, Rzeszów, Poland

**Keywords:** multiple sclerosis, religiosity, life satisfaction, meaning in life, Polish patients

## Abstract

**Introduction:**

The complexity of the associations between religiosity and indicators of well-being suggests the presence of a mediating mechanism. Previous studies indicate that religion may influence subjective well-being because it helps to find meaning and purpose. Therefore, the aim of our study was to examine the mediating role of the presence and search dimensions of meaning in life in the relationship between religious meaning system and life satisfaction in patients with multiple sclerosis (MS).

**Methods:**

This cross-sectional study included 600 MS patients recruited from Poland who completed the Satisfaction with Life Scale (SWLS), the Religious Meaning System Questionnaire (RMS) and the Meaning in Life Questionnaire (MLQ). Model 6 of Hayes PROCESS was used to test the hypotheses.

**Results:**

The results of our research indicate that there was a significant indirect effect of religious meaning system on life satisfaction through the presence of meaning in life. The specific indirect effect of religious meaning system on life satisfaction through searching for meaning in life was not significant.

**Discussion:**

The results of our study are relevant because they show that religion as a meaning system is positively related to the presence of meaning in life, which in turn positively predicts life satisfaction. This is particularly important in the case of incurable illness, where finding meaning in life is one of the natural stages of adaptation. By incorporating these findings into mental health practice, professionals can enhance the holistic well-being of people coping with MS and contribute to a more comprehensive and effective approach to mental health care.

## 1 Introduction

Multiple sclerosis (MS) is a chronic disease of the central nervous system characterized by multiple motor, affective, sensory and cognitive deficits. These deficits include: muscle spasms, stiffness and weakness; pyramidal, sensory and cerebellar dysfunction; pain; fatigue; bladder, bowel and sexual dysfunction; and mood disturbances (1). Although MS is not fatal, over time it leads to disability and reduced life function. Current treatments for MS can slow the progression of the disease, but no cure has been found. The course of the disease varies from patient to patient. Because MS is characterized by different dynamics and a wide range of symptoms, it is often uncontrollable and unpredictable. Periodic relapses and remissions, as well as the potential side effects of treatment and the sense of uncertainty following diagnosis, increase the emotional cost to patients of coping with the disease (2).

### 1.1 Religiosity and life satisfaction in MS

Research has shown that religiosity is an effective coping mechanism for people coping with chronic illnesses such as MS (3–6). In particular, studies highlight that people with MS use religious strategies to improve their mental functioning and overall life satisfaction. Life satisfaction is a complex concept that involves the assessment of different domains of functioning, and researchers often use terms such as quality of life and well-being interchangeably due to their overlapping nature. In essence, it reflects an individual’s subjective assessment of the quality and contentment of different aspects of their lives, providing a holistic perspective on their overall sense of fulfillment and happiness.

Consistent positive associations between spirituality/religiosity and life satisfaction among Canadians with neurological conditions, including MS, have been reported in cross-sectional studies (7). Giovanolli et al. (8) reported similar findings and found that spiritual and religious beliefs predicted quality of life in people with neurological disorders. Another qualitative study involving 13 people with MS found that adaptation to the disease is influenced by a number of factors, with religion/spirituality playing an important role (9). Wade and colleagues (10) further supported these findings by suggesting that individuals with strong existential spiritual beliefs experienced greater happiness in the face of neurological illness, including MS. These findings highlight the complex relationship between religiosity, spiritual beliefs and various dimensions of life satisfaction, and shed light on their constructive role in the adjustment of individuals coping with chronic diseases such as MS.

Despite existing evidence, the current state of knowledge about the relationship between religiosity and adjustment to MS remains uncertain. Some studies have contradicted the positive associations found in previous research. For example, Makros and McCabe (11) found no significant association between religiosity and psychological adjustment or quality of life in people with MS. Similarly, Bussing et al. (12) found that faith as a resource was not significantly associated with mood, disease progression or life satisfaction in young people with MS. These discrepancies in findings may be due to several factors.

First, religiosity has been defined in different ways in different studies. Some researchers have measured religious involvement, others have distinguished between religiosity and spirituality or between extrinsic religious behavior and intrinsic experiences of faith, and some authors refer to religiosity as a belief system or level of religious practice (13–15). These constructs are often difficult to compare because researchers using them measure very different aspects of religiosity, which can lead to differences in results. As shown by Wade et al. (10), different components of spirituality have different relationships with quality of life in neurological patients.

Second, cultural issues also play an important role. Bussing et al. (12) compared the results of American and German studies (societies with different levels of religiosity) and found that the importance of spirituality in coping with MS may differ with respect to cultural and specific religious issues. Furthermore, religious people have higher rates of subjective well-being in religious nations, whereas non-religious people have higher rates of subjective well-being in non-religious nations (16, 17). Poland is one of the most religious countries in Europe (18), with over 90% of the population belonging to Christianity, the highest percentage on the continent. Nearly 50% of the population in Poland have declared that they practice their religion regularly (19). Therefore, taking into account its cultural significance in Poland, religiosity can be considered as an effective way of coping with chronic diseases and related problems.

### 1.2 Religious meaning system

In our study, we analyzed religiousness in terms of the religious meaning system (20, 21), which can be defined as an idiosyncratic system of concepts related to the sacred and having meaning-oriented references to self, other people and the world (22). Religiousness in terms of the religious meaning system is based on shared observations that religion enables interpretation and clarification of reality in the categories of meaning and purpose (22). Being diagnosed with an illness creates uncertainty and forces a person to seek ways of dealing with the unknown (23). The religious meaning system, when viewed as a guiding philosophy of life, provides a unique perspective on difficult life events and motivates people to cope with the challenges of the disease by setting goals and values. It can also help to discover the hidden meaning of events, even those that are very complex and difficult to accept, such as the diagnosis of MS. In such a paradigm, religiosity could be a critical cognitive resource for someone coping with an unpredictable disease that changes its course over time (24).

A growing body of research suggests that, in many populations, the relationship between religiosity and broadly defined life satisfaction may be mediated by other important psychosocial factors. These factors include social support (16, 25), prosocial behavior (26), coping (27, 28), hope (29–32), positive orientation (25, 33–35), and meaning in life (14, 22, 31, 32, 36–38). Of these, meaning in life may play a specific mediating role in the relationship between religiosity and adjustment outcomes in MS.

### 1.3 Meaning in life

Although meaning in life has been conceptualized in many ways, it is generally referred to as people’s coherent understanding of themselves and their life experiences, and their possession of a purpose, mission or overarching goal in life (39–41). Steger et al. (42) proposed two dimensions of meaning in life: the search for meaning in life (motivational aspect), which refers to the degree to which people engage in the search for meaning in life; and the presence of meaning in life (cognitive aspect), which refers to the degree to which individuals perceive their lives as significant and meaningful. Previous research has shown that different dimensions have different levels of importance for different psychological factors (43). The presence of meaning in life is often considered a stronger correlate of human well-being than the search for meaning in life (44, 45). A review of 147 studies by Li et al. (45) found that the presence of meaning in life was positively and moderately correlated with subjective well-being indicators (0.418), whereas the search for meaning in life was negatively and weakly correlated with these measures (−0.121). However, some studies have reported conflicting results, suggesting a positive role for the search for meaning in anxiety, positive affect or life satisfaction (46, 47).

Culture and differences in the groups studied appear to be key factors in the lack of clear results. Nevertheless, the literature provides ample evidence of the significant association of meaning with religiosity and subjective and psychological well-being, as well as the mediating role of meaning in this relationship (36–38, 48).

Certainly, the presence or search for meaning in life may be an important factor in coping with illness and improving well-being, as evidenced by research (49, 50). The presence of meaning in life allows patients to make sense of their difficult times, gain a sense of control and cope constructively with physical and mental challenges (38). Finding meaning and purpose helps maintain hope and provides motivation for treatment (20). This is particularly important for people diagnosed with illnesses characterized by an unpredictable and uncertain course. Meaning making enables people to reduce their sense of uncertainty and to regain a vision of current events and their own lives, making them coherent and understandable (51).

### 1.4 Religiosity and meaning in life

A well-established religious belief system can contribute to a sense of meaning in life (52). Shiah et al. (53) reported that religious people have a higher awareness of coherence and purpose in life than non-religious people. People develop religious beliefs and engage in religious activities to make sense of complex and incomprehensible events (48). Religion influences individuals by modifying their ways of thinking, their beliefs about themselves and the world, and their understanding of life events and experiences, which in turn can initiate the meaning-making process (54). Findings suggest that the greater a person’s religious meaning system, the greater the association with life meaning and life satisfaction (22). In other studies of late adolescents, higher religiosity, conceptualized as religious meaning system, was associated with stronger meaning-making, which in turn was associated with higher levels of life satisfaction and lower levels of negative affect (38). People who are more religious are predisposed to derive personal meaning and positive evaluations from their religious beliefs, which in turn enable them to cope with adversity and life stress (22, 55).

The complexity of the associations between religiosity and indicators of life satisfaction suggests the presence of a mediating mechanism. Previous studies suggest that religion may influence life satisfaction because it helps to find meaning and purpose (14, 22, 36–38). We would like to test this hypothesis in a group of people with MS. Therefore, in this study, we examined the mediating role of the presence and search dimensions of meaning in life in the relationship between religious meaning system and life satisfaction in people with MS. In addition, Krok’s research (22) has shown that for religion to play a positive role in overall life satisfaction, the meaning of life should have a presence rather than a search nature. In other words, individuals need to have a belief that their lives form a coherent and purposeful whole, enabling them to perceive their own lives as meaningful and significant. In line with these findings, we assumed that the presence of meaning in life would be a stronger mediator than the search for meaning in life.

## 2 Methods

### 2.1 Study population and design

The cross-sectional study included 600 people with MS, recruited between January and December 2019 from nine Polish centers involved in the diagnosis and treatment of MS (Białystok, Końskie, Międzylesie, Rzeszów, Sandomierz, Szczecin, Warszawa (2 centres), and Zabrze). Inclusion criteria for the study were: (1) age between 17 and 70 years; (2) clinically definite MS according to the 2017 or 2010 McDonald criteria (56); and (3) informed consent to participate in the study. Exclusion criteria included: (1) advanced disease that prevented participation in the study, such as cognitive or speech impairment; and (2) coexistence of neoplastic disease. Subjects were recruited during their attendance at the rehabilitation clinic for a pre-arranged consultation with their physician. Before entering the doctor’s office, each patient was invited by a member of the research team, who explained the study in detail and ensured understanding. Patients who agreed to participate were then asked to provide written informed consent. Adolescents under 18 years of age participated in the study with the consent of their parents or legal guardians. Participants were assured of the confidentiality of their data. The research instruments were administered in person by members of the research team in a dedicated, quiet room, and the entire process took approximately 30 min. Although the questionnaires were checked for completeness immediately on return and respondents were asked to fill in any unintended gaps, 18 of the 618 people who completed all the questionnaires did not provide complete data and were therefore excluded from the study. The study protocol was approved by the Bioethics Committee of the Institute of Psychology, University of Szczecin, Poland (KB 13/2021, 20.05.2021). The study was conducted in accordance with the tenets of the Declaration of Helsinki.

The socio-demographic and clinical information of the participants was collected by questionnaire prior to the assessment and is presented in Table 1.

**Table 1:** Demographic and clinical characteristics of study population.

Variable	Group	*N*	%
Sex	Female	436	72.7
	Male	164	27.3
Educational level	Elementary	20	3.3
	Secondary	216	36
	High	278	46.3
	Vocational	86	14.3
Marital status	Married	399	66.5
	Divorced	41	6.8
	Never married	150	25
	Widowed/separated	10	1.7
Place of residence	Country	227	37.8
	Town <10,000 inhabitants	88	14.7
	Town <100,000 inhabitants	129	21.5
	Town >100,000 inhabitants	156	26
Residence	Flat	233	38.8
	Multi-family house	36	6
	Semi-detached house	23	3.8
	Single-family house	308	51.3
Living situation	Live with spouse and children	310	51.7
	Live with spouse	103	17.2
	Live with children	34	5.7
	Live with parents	87	14.5
	Live alone	66	11
Disease course	With relapses	254	42.3
	Stable without relapses	239	39.8
	Slowly progressive	107	17.8
Ambulation	Independent	484	80.7
	Independent with device	92	15.3
	Requires assistance of one other person	24	4
Financial status	Tragic	7	1.2
	Very bad	5	0.8
	Bad	49	8.2
	Average	277	46.2
	Good	218	36.3
	Very good	44	7.3
Smoking status	Yes	87	14.5
	No	513	85.5

### 2.2 Measures

Three research instruments were used to measure religious meaning system, meaning in life and life satisfaction.

The Religious Meaning System (RMS) questionnaire (22) was used to assess levels of religious meaning system. The questionnaire conceptualizes religiosity as a cognitive and motivational system that allows people to understand and interpret their personal experiences in terms of orientation and meaning. The RMS questionnaire consists of 20 items scored on a 7-point Likert scale ranging from 1 (strongly disagree) to 7 (strongly agree). A high score represents a higher level of religiosity. The questionnaire has two scales: religious orientation (10 items) and religious significance (10 items). Because of their strong correlation (*r* = 0.88, *p* < 0.01), we decided to use the religious meaning system as a single dimension without splitting it into religious orientation and religious meaning. The psychometric properties of the RMS were very good. The overall Cronbach’s alpha was equal to *α* = 0.92.

To measure meaning in life, we used the Meaning in Life Questionnaire (MLQ) (42), adapted into Polish by Kossakowska, Kwiatek, and Stefaniak (57), which assesses meaning in life as the stated meaning and significance felt about the nature of one’s being and existence. The MLQ consists of 10 items that measure two dimensions of meaning in life: (1) presence of meaning (the extent to which respondents feel that their lives have meaning) and (2) search for meaning (the extent to which respondents strive to find meaning and understanding in their lives). Each item is rated on a 7-point Likert scale ranging from 1 (completely true) to 7 (completely false). The MLQ has been shown to have high internal consistency (42). In the present study, the presence of meaning subscale (Cronbach’s alpha = 0.88) and the search for meaning subscale showed very good coefficients (Cronbach’s alpha = 0.87).

To measure life satisfaction, we used the Polish version of the Satisfaction with Life Scale (SWLS) developed by Diener et al. (58, 59). The SWLS is a short instrument with only five items measuring global cognitive judgments of life satisfaction. Participants are asked to indicate how much they agree or disagree with each of the five items on a 7-point scale ranging from 7 (strongly agree) to 1 (strongly disagree). In this study, the SWLS scale showed high internal consistency (Cronbach’s alpha = 0.9).

### 2.3 Statistical analyses and research model

Statistical analyses were performed using IBM SPSS statistical software (version 27.0). Model 6 of the PROCESS macro by Hayes (60) was used to test the relationships between variables. To test the significance of the indirect effects, we used the bootstrapping method with 5,000 subsamples and 95% bias-corrected confidence intervals (CIs). If the CI does not include zero, the parameter is considered significant. The effect size for the specific indirect effect was calculated as a fully standardized indirect effect (61).

In order to confirm whether the observed effects are statistically significant in each individual case, it is required that the bootstrapped CI does not include zero (60).

The research model assumed that the presence of meaning in life and the search for meaning in life mediate the relationship between religious meaning system and life satisfaction.

## 3 Results

### 3.1 Preliminary results

Descriptive statistics in the sample of patients diagnosed with MS are shown in Table 2.

**Table 2:** Descriptive statistics in sample of patients diagnosed with multiple sclerosis.

	SWLS	MLQP	MLQS	RMSQ
Mean	20.71	23.55	21.19	61.25
Standard deviation	5.99	5.32	4.86	23.81
Skewness	−0.21	−0.34	−0.27	−0.55
Kurtosis	−0.16	0.3	0.26	−0.09
Minimum	5	5	5	0
Maximum	35	35	35	105
Reliability Cronbach’s α	0.90	0.88	0.87	0.92
VIF		1.25	1.26	1.01

SWLS, Satisfaction With Life Scale; MLQP, Meaning in Life Questionnaire – presence; MLQP, Meaning in Life Questionnaire – searching for; RMSQ, Religious Meaning System Questionnaire; VIF, Variance Inflation Factor.

As a first step, we conducted a correlation analysis of the measured variables. Correlations showed interdependencies between the variables included in the mediation model, i.e., religious meaning system, life satisfaction, presence and search for meaning in life. Correlations were also found between the model variables and variables such as age, disease duration, financial status, place of residence, disease progression, and walking ability. Detailed results of the bivariate correlations are presented in Table 3.

**Table 3:** Bivariate correlations in sample of patients diagnosed with multiple sclerosis.

Variables	(1)	(2)	(3)	(4)	(5)	(6)	(7)	(8)	(9)	(10)	(11)
(1) Life satisfaction	1										
(2) Presence of meaning	0.72**	1									
(3) Search for meaning	0.58**	0.65**	1								
(4) Religious meaning system	0.16**	0.28**	0.21**	1							
(5) Age	−0.02	0.05	−0.06	0.14**	1						
(6) Educational level	0.03	0.04	0.03	0.04	0.04	1					
(7) Illness duration	−0.03	0.04	0.04	0.08*	0.08	0.01	1				
(8) Place of residence	0.10*	0.06	0.04	−0.21**	−0.11*	−0.04	0.04	1			
(9) Financial status	0.41**	0.26**	0.16**	−0.02	−0.11*	0.06	−0.02	0.17**	1		
(10) Disease course	0.04	0.05	−0.01	0.10*	0.11**	0.09*	0.10*	−0.07	−0.01	1	
(11) Ambulation	−0.22**	−0.20**	−0.16**	−0.01	0.27**	−0.03	−0.01	−0.08*	−0.28**	0.18**	1

*p ≤ 0.05, **p ≤ 0.01.

### 3.2 Mediation analysis

**Figure 1:**
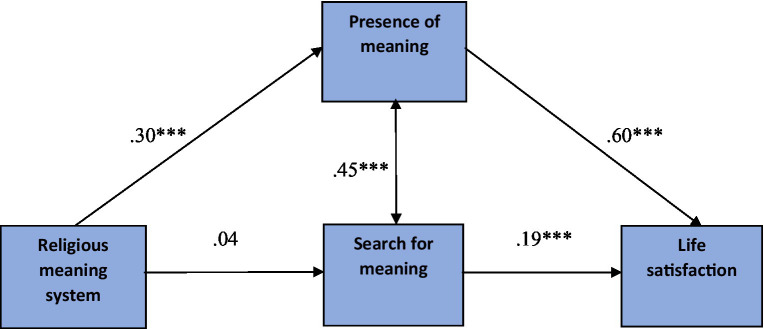
Presence of meaning as a mediator between religious meaning system and life satisfaction. The standardized regression coefficients are presented. *p < 0.05, **p < 0.01, ***p < 0.001.

The standardized parameters for the mediation relationship are shown in Figure 1.

The overall effect of religious meaning system on life satisfaction was significant (*β* = 0.18; 95% CI [0.024, 0.062]; *p* < 0.001) and consisted of a significant overall indirect effect (*β* = 0.22; 95% CI [0.137, 0.306]; *p* < 0.001) and a nonsignificant direct effect (*β* = −0.04; 95% CI [−0.024, 0.003]; *p* = 0.124).

The direct effect of the religious meaning system on the presence of meaning in life was statistically significant (95% CI [0.051, 0.086], *p* < 0.001, *β* = 0.30), in contrast to the direct effect of the religious meaning system on the search for meaning in life, which was not significant (95% CI [−0.005, 0.024], *p* = 0.193, *β* = 0.04). The direct effect of having meaning in life on life satisfaction was also statistically significant (95% CI [0.554, 0.706], *p* < 0.001, *β* = 0.60), as was the direct effect of searching for meaning in life on life satisfaction (95% CI [0.139, 0.292], *p* < 0.001, *β* = 0.19).

Regarding the specific indirect effects, there was a significant indirect effect of religious meaning system on life satisfaction through the presence of meaning in life (*β* = 0.16, 95% CI [0.098, 0.221]). The specific indirect effect of religious meaning system on life satisfaction through the search for meaning in life was not significant (*β* = −0.01, 95% CI [−0.008, 0.025]). The most complex indirect effect tested was the cascading effect of religious meaning system on life satisfaction through the sequential path of presence of meaning in life – searching for meaning in life was significant (*β* = 0.04, 95% CI [0.020, 0.063]).

Among the controlled variables, only a statistically significant correlation was found between gender and life satisfaction (*r* = 0.05, *p* = 0.048), between disease duration and life satisfaction (*r* = −0.06, *p* = 0.031), and between financial status and life satisfaction (*r* = 0.22, *p* < 0.001).

## 4 Discussion

The main aim of this study was to determine whether the presence and search dimensions of meaning in life mediate the relationship between religious meaning system and life satisfaction in patients with MS. In a sample of Polish patients diagnosed with MS, religion as a meaning system was positively related to the presence of meaning in life, which in turn positively predicted life satisfaction. To our knowledge, this is the first study to examine the relationships between these variables in people with MS.

Our findings are consistent with those of previous studies showing the beneficial role of purpose in life. Previous studies have shown that meaning in life mediates the relationship between religiousness and life satisfaction (37), religion and well-being (36), religious orientation and subjective well-being (14), and religious coping and personal well-being (48).

However, all of these studies were conducted on non-clinical samples. One exception is the study by Krok (62), which showed that religious meaning system and life meaning accurately predicted the psychological well-being of cancer patients. Some studies have made assumptions about the different ways in which people with life-threatening illnesses such as cancer, heart disease or AIDS use religious resources compared to those with chronic disabilities. The proposed distinction suggests that individuals with life-threatening illnesses may use religious resources with a focus on preparing for death, whereas those with chronic disabilities may use them to prepare for a life that spans decades and is characterized by significant physical and neuropsychological impairments (63). This line of research is particularly relevant in the context of MS. Reports from the literature highlight notable differences in religious attitudes between patients with MS and those with cancer. Specifically, patients diagnosed with MS tend to have significantly lower levels of religious attitudes compared to their counterparts with cancer (64, 65). These findings highlight the importance of recognizing the diversity of responses to chronic health conditions and the potential differences in the ways in which individuals draw on religious resources depending on the nature and prognosis of their condition. Further exploration of these differences may contribute to a broader understanding of the role of religion in coping with and adapting to different health challenges.

Based on our study, we can conclude that the religious meaning system plays an important role as a resource in MS and is associated with life satisfaction through the presence of meaning in life. Chronic illness pushes patients to search for meaning in life and religiosity can be an important source of this meaning. Thus, we can confirm Krok’s (22) suggestion that people who have a higher level of religious meaning system may be predisposed to derive positive evaluations and greater meaning in life from their religious beliefs in order to increase their life satisfaction.

Regarding the search dimension of meaning in life, our results do not confirm a relationship between this variable and life satisfaction, which contradicts the findings of some previous studies. Some authors have observed a negative relationship between the search for meaning in life and well-being (66–68), while others report a contradictory relationship (47, 69, 70). These differences can be explained by different theoretical approaches, which are dominated by two opposing views. According to the first view, meaning-seeking dimensions of life may serve as a resilience factor that helps people experiencing negative life events to seek new opportunities, re-understand and reorganize past experiences, and overcome challenges, which in turn leads to better adjustment and positive changes in well-being (45, 71, 72). On the other hand, the search for meaning in life may result from a lack of purpose in life and associated frustration. Put simply, in times of crisis and stress, searching for meaning in life may be a valuable coping strategy, but it may be destructive for those who are not facing frustrating situations, as it may represent a loss of life goals (45). Thus, the life situation determines whether the search for meaning in life has a positive or negative effect on life satisfaction. Our results do not support either of these approaches, as there was no relationship between the search dimension of meaning in life and life satisfaction in people with MS.

The reasons for the inconsistent findings may also be due to cultural factors. Fischer and colleagues (73) showed that the benefits of the presence of meaning may be similar across cultures, while the search for meaning may be culturally specific, producing different effects on psychological well-being. Kossakowska, Kwiatek, and Stefaniak (57) included the cultural factor in the Polish version of the MLQ. The results of the Polish research are not clearly similar to those of the American research (42). It was found that the search for meaning in life is not considered pleasant by the American population. It seems to be negatively correlated with well-being (42) and to bring chaos into life. On the other hand, in Poland (as well as in Japan) the search for meaning in life does not have such negative connotations (57, 74). It is possible that, in addition to cultural conditioning, the relationship between the search for meaning and life satisfaction depends on the research sample. The specificity of a sample may be a moderator in this relationship. For example, in a sample of Sexaholics Anonymous from Poland, the search for meaning was negatively related to life satisfaction (75).

Taking into account the specificity of the study group, the course of MS is highly unpredictable and is usually characterized by relapses and periods of symptom remission. Uncertainty about treatment outcomes and the high rate of disability progression, as well as the side effects of the therapies used, make it difficult for patients to adjust psychologically to the disease state and have a significant impact on their daily functioning, mental health (76, 77), quality of life (78) and ability to cope (79). The search for meaning in life dimension represents the motivational aspect of meaning in life, which requires commitment and increased activity (43). The dynamics of MS, uncertainty and the need to respond to changing symptoms of the disease engage patients to such an extent that there is no room for other activities. This is, of course, an assumption that requires more in-depth analysis and comparative studies with patients with different disease characteristics, but there are reports that patients with MS are significantly less engaged in various forms of spiritual practice than cancer patients (65).

The results of our study are relevant because they show that religion as a meaning system is positively related to the presence of meaning in life, which in turn positively predicts life satisfaction. This is particularly important in the case of incurable diseases, when finding meaning in life is one of the natural stages of adaptation (20). Professionals can take advantage of the process of finding meaning in living with the disease, as it is positively associated with life satisfaction. An effective way to target this important clinical variable might be to address meaning-making efforts in therapeutic settings, perhaps through cognitive restructuring techniques. Religious people are more likely to use meaning-making strategies such as positive reappraisal coping, which involves looking for and emphasizing the positive aspects and implications of situations (80). The therapist can help through dialog to focus on the aspects of a stressful situation that can lead to growth and opportunity. Drawing on their religious beliefs, through the process of cognitive reconstruction to fit the illness into one’s life story, individuals may value a serious illness as an opportunity for development. Supporting the reinterpretation of the diagnosis as a challenge or opportunity allows for a greater sense of control over the illness and its treatment (81). The existing religious system of meaning can be useful in achieving this goal, particularly for communities that see religion as a critical element in shaping worldviews.

There are some limitations to this study. The first is the way we assessed religiosity. We assumed that the religious meaning system reflected those characteristics of religiosity that play an important role in coping with the disease, but we cannot exclude other measures of religiosity assessment that should be evaluated in future studies for a more thorough analysis of the role of religious factors in life satisfaction among people with MS. Second, because our results are based on a cross-sectional sample, they cannot distinguish temporal order, which limits our ability to infer directionality and causality in the relationships. Thirdly, we did not control for variables such as disease severity (although this is often related to disease duration, which was controlled for) or cognitive impairment, a common effect of MS that may affect adequate self-assessment. Finally, religion in Poland is dominated by Catholic Christians, so our findings cannot be generalized to multi-religious societies.

Despite these limitations, our results indicate an important mediating role of the presence dimension of meaning in life in the relationship between religious meaning system and life satisfaction among Polish patients with MS. In other words, the religiosity of MS patients is associated with life satisfaction through the presence of meaning in life, which may be an important resource for coping with the difficulties of everyday life. By incorporating these findings into psychiatric practice, professionals can enhance the holistic well-being of people coping with MS and contribute to a more comprehensive and effective approach to mental health care.

## Data availability statement

The original contributions presented in the study are included in the article/supplementary materials, further inquiries can be directed to the corresponding author.

## Ethics statement

The studies involving humans were approved by Bioethics Committee of the Institute of Psychology at University of Szczecin, Poland (KB 13/2021, 05.20.2021). The studies were conducted in accordance with the local legislation and institutional requirements. The participants provided their written informed consent to participate in this study. Written informed consent was obtained from the individual(s) for the publication of any potentially identifiable images or data included in this article.

## Author contributions

MWi: Conceptualization, Data curation, Formal analysis, Methodology, Resources, Supervision, Writing – original draft, Writing – review & editing. MWn: Conceptualization, Data curation, Formal analysis, Methodology, Resources, Writing – original draft, Writing – review & editing. WB: Conceptualization, Data curation, Formal analysis, Resources, Supervision, Writing – original draft, Writing – review & editing. MS: Methodology, Resources, Writing – original draft. MŻ: Data curation, Formal analysis, Investigation, Resources, Writing – original draft. PS: Data curation, Formal analysis, Investigation, Resources, Writing – original draft. KK-T: Formal analysis, Investigation, Resources, Writing – original draft. JT: Formal analysis, Investigation, Resources, Writing – original draft. AC: Formal analysis, Investigation, Resources, Writing – original draft. AK: Formal analysis, Resources, Writing – original draft. BZ-P: Formal analysis, Resources, Writing – original draft. HB-P: Formal analysis, Resources, Writing – original draft. KK-B: Formal analysis, Resources, Writing – original draft. NM: Formal analysis, Resources, Writing – original draft. MA-S: Writing – original draft. AS: Formal analysis, Resources, Writing – original draft. ZJ: Formal analysis, Resources, Writing – original draft. AR: Formal analysis, Resources, Writing – original draft. MR: Formal analysis, Resources, Writing – original draft. RS: Formal analysis, Resources, Writing – original draft. ZK: Formal analysis, Resources, Writing – original draft. BL: Formal analysis, Resources, Writing – original draft. APe: Formal analysis, Resources, Writing – original draft. MP: Formal analysis, Resources, Writing – original draft. APo: Conceptualization, Formal analysis, Funding acquisition, Investigation, Methodology, Project administration, Resources, Supervision, Writing – original draft, Writing – review & editing.
